# Scale for Assessing Understanding of Benefits and Risks of Using Complementary Therapies in Diabetes Management: Psychometric Evaluation

**DOI:** 10.1097/jnr.0000000000000717

**Published:** 2026-01-09

**Authors:** Hsiao-Yun CHANG, Yu-Yao HUANG, Chia-Lun LO

**Affiliations:** 1 Department of Nursing, Chang Gung University of Science and Technology, Taoyuan, Taiwan; 2 Department of Internal Medicine, Division of Endocrinology and Metabolism, Chang Gung Memorial Hospital, Taoyuan, Taiwan; 3 Department of Medicine, College of Medicine, Chang Gung University, Taoyuan, Taiwan; 4 Department of Health-Business Administration, Fooyin University, Kaohsiung, Taiwan

**Keywords:** instrument development, benefit, risk, complementary therapies (CTs), diabetes

## Abstract

**Background:**

The lack of a comprehensive assessment tool to evaluate patient understanding of the benefits and risks of complementary therapies (CTs) in diabetes management may lead to the unsafe use of CTs alongside conventional treatments, increased risk of misinformed decision-making, potential adverse interactions, and compromised health outcomes.

**Purpose:**

In this study, an instrument was developed to assess patient understanding of the benefits and risks of using CTs in diabetes management and its psychometric properties were evaluated.

**Methods:**

A two-phase design, including scale development and psychometric validation, was used. In Phase 1, the initial scale items were revised and confirmed by a panel of experts for content validity. In Phase 2, a cross-sectional survey was conducted to assess the scale’s psychometric properties, including reliability and validity analyses. A sample of 307 outpatients with diabetes who had used CTs all completed a sociodemographic, clinical characteristics, and CT usage data sheet, as well as the Understanding the Benefits-Risks of CT Use in Diabetes Scale and the Diabetes Empowerment Scale. The developed scale was validated using exploratory and confirmatory factor analyses that used structural equation modeling to confirm construct validity, Person correlation to confirm criterion-related validity, and Cronbach’s α coefficient to confirm reliability.

**Results:**

The initial 16-item Understanding the Benefits-Risks of CT Use in Diabetes Scale was assessed as having a content validity index of .88. Factor analyses reduced the scale to 15 items in four dimensions, including the patient’s medical condition for CT use (four items), the benefit-risk assessment of CTs use (four items), the suitability of CT use (five items), and the support from health care professionals (two items). The model met all goodness-of-fit indices (GFI=.90, AGFI=.90, CFI=.94, NFI=.926, RMSEA=.08, and χ^2^/*df*=3.14). The reliability analysis indicated good internal consistency (α=.912) and correlation with the Diabetes Empowerment Scale (*r*=.425).

**Conclusions/Implications for Practice:**

The Understanding the Benefits-Risks of CT Use in Diabetes Scale offers valuable insights for both patients and health care professionals. By providing a comprehensive assessment of patient knowledge and awareness of their CT use, this tool helps health care professionals identify gaps in patient understanding, tailor patient education, and ensure safe and effective integration of CTs into diabetes management programs.

## Introduction

Diabetes mellitus, a chronic metabolic disorder characterized by hyperglycemia, poses an escalating global health burden and affects millions of individuals worldwide (Sun et al., 2022). The management of diabetes requires a multifaceted approach encompassing lifestyle modifications, pharmacotherapy, and, more recently, the integration of complementary therapies (CTs) into conventional treatment strategies (Alzahrani et al., 2021). CTs encompass a diverse range of health care practices, products, and systems that fall outside conventional medical practice and often appeal to patients seeking holistic and integrative approaches to their disease management (Mori et al., 2023).

The rising popularity of CTs in diabetes management has prompted a critical need to understand the potential benefits and risks associated with their use to ensure safe and effective integration with conventional diabetes treatments (H. Y. A. Chang, 2017). However, most existing scales overlook patient preferences, knowledge, beliefs, and attitudes regarding CT use (Alzahrani et al., 2022; Tehrani et al., 2022), and the literature lacks a valid and comprehensive scale for assessing patient understanding of the benefits and risks of using CTs in diabetes management. H.-Y. Chang et al. (2021) and H.-Y. Chang, Yang, et al. (2023) used a Delphi–analytic hierarchy process approach and text mining algorithms to identify the critical factors and consensus-driven algorithms used by health care professionals to assess and manage the risk-benefit profile of complementary and integrative therapies in diabetes care. Despite these valuable insights, there is a significant need for a validated and comprehensive tool to assess patient understanding of the benefits and risks associated with the use of CTs. This understanding is essential to enhancing patient-provider communication, personalizing treatment plans, ensuring the safe integration of therapies, and promoting informed decision-making in diabetes management. To address this gap, this paper was designed to develop and validate a benefits-risks scale that provides relevant insights into patient understanding, supports a patient-centered approach, and promotes informed decision-making and patient safety with regard to CT use in diabetes care.

### Background

#### The benefits and risks of using CTs in diabetes care

CTs used by patients with diabetes encompass a diverse array of approaches, including herbal supplements, acupuncture, mind-body therapies, religious and spiritual healing, homeopathy, meditation, massage, ayurveda, chiropractic, energy therapies, specific diet, and yoga (Alzahrani et al., 2021). Explorations of the potential benefits of these modalities in diabetes management have demonstrated improvements in glucose control, reductions in diabetes-related complications, and enhanced quality of life (Meuffels et al., 2022; Setiyorini et al., 2022). For instance, herbs and supplements, such as chromium, n-3 polyunsaturated fatty acids, vitamin D, zinc, magnesium, *Berberis aristata/Silybum marianum*, fenugreek seed, bitter melon, cinnamon, and chamomile tea, have shown promising effects on glycemic control as well as antioxidant properties that are of potential benefit to patients with diabetes (Behrouz et al., 2020; Setiyorini et al., 2022). In addition, acupuncture has been investigated for its impact on hypoglycemic outcomes in diabetes, revealing improvements in fasting blood glucose levels and insulin resistance (Li et al., 2022). Similarly, mind-body practices, particularly yoga, have demonstrated positive effects on glycemic control and stress management outcomes among patients with diabetes (Sanogo et al., 2023).

Despite the mounting evidence supporting the potential benefits of using CTs in diabetes management, it is essential to consider the potential associated risks, including adverse effects, interactions with conventional medications, and the possibility of delayed medical treatment due to an over-reliance on CTs (H.-Y. Chang, Yang, et al., 2023). For instance, several CTs that use herbs, supplements, vitamins, homeopathy, and dietary interventions have been found to have adverse effects and to interact readily with conventional medications (Stub et al., 2022; Thikekar et al., 2021). Understanding the potential benefits and risks of CT use will facilitate informed decision-making for health care professionals and patients, facilitating the making of well-balanced choices about the integration of CTs into conventional treatment regimens.

#### Challenges in assessing the risk of integrating CTs into diabetes care

Integrating CTs into conventional health care requires careful consideration of the potential risks. Adverse effects resulting in unintended side effects or reduced treatment efficacy due to interactions with prescription medications have been highlighted in the literature, with some CT therapies potentially inducing allergic or other adverse reactions in some patients (Thikekar et al., 2021). Also, surveys have reported that some individuals take CT and conventional medications separately or even delay taking conventional medicines to avoid herb–drug interactions (Adeniyi et al., 2021; Alzahrani et al., 2021). Without proper coordination or oversight from health care professionals, missed opportunities for comprehensive and optimized treatment plans may compromise patient safety and health outcomes (H.-Y. Chang et al., 2021).

Incorporating CTs into conventional health care warrants vigilant attention to other potential risks beyond the adverse effects and interactions associated with the concurrent use of prescription medications (Stie et al., 2020). Patients must consider the suitability of CT use in light of their health conditions and be aware of the limited evidence and quality control associated with CTs. Moreover, the absence of standardized dosages and formulations in CTs, as well as CT-related misinformation and fraudulent practices, necessitate careful consideration (H.-Y. Chang, Mao, et al., 2023). To address these complexities, one research team developed a multicriteria algorithm to guide optimal decision-making by health care professionals for managing CT use in patients with diabetes (H.-Y. Chang et al., 2021). This algorithm encompasses diverse factors, including patient data, CT applicability, product attributes, usage safety, and institutional culture, with the goal of comprehensively assessing the benefits/risks of CT use to support a patient-centered approach, informed decision-making, and the integration of CTs into diabetes care.

#### Current CT use assessment tools

Several assessment tools have been developed to evaluate pattern usage, knowledge, beliefs, attitudes toward, behavior, satisfaction, and outcomes related to CTs in different patient populations (Tangkiatkumjai et al., 2020). For instance, the International Complementary and Alternative Medicine questionnaire has been used widely to investigate patient use of CTs, the reasons for CT use, and the perceived benefits of CT use (Quandt et al., 2009). In addition, scales designed to assess patient CT-related beliefs, attitudes, expectations, and effectiveness perceptions include the Holistic CAM (complementary and alternative medicine) Questionnaire (Hyland et al., 2003), CAM Health Belief Inventory (Bishop et al., 2005), and Complementary and Alternative Medicine Health Belief Questionnaire (Lie & Boker, 2004). These assessment tools are designed primarily to gather insights into patient preferences, beliefs, experiences, and perceived outcomes related to CT use. However, they may not adequately assess the comprehensive understanding of patients regarding both the benefits and risks associated with CT use.

Similar tools for assessing patient understanding of the benefits and risks of using CTs include the Michigan State University Complementary and Alternative Medicine Health Literacy Scale (Shreffler-Grant et al., 2014) and the questionnaire to assess what guides decision-making in patients with cancer when comparing conventional medicine and CTs (Hack et al., 2023). These instruments offer insights into a patient’s ability to obtain, understand, and apply information about CTs, their level of engagement in the decision-making process, and their satisfaction with treatment decisions made. Although these tools provide important general information regarding patient knowledge and engagement, they do not capture all of the relevant information or the disease-specific considerations required to assess the potential risks and safety concerns related to individual CTs in response to conventional treatments. Complexities arise when considering CT use in the treatment of specific diseases such as diabetes due to the potential interactions with conventional diabetes medications and the need to thoroughly assess the impact of CTs on glycemic control and diabetes-related complications.

To address this gap, designing an assessment tool that captures more comprehensive information on the safety and risks associated with each CT is of clinical importance. A holistic assessment tool that provides patients with an understanding of the benefits and risks of CTs will also help health care professionals engage in better-informed decision-making discussions about integrating CTs into diabetes management plans, leading to better treatment outcomes and improved health results. Therefore, the aim of this study was to develop a scale for patients with diabetes able to measure their understanding of the benefits and risks of CT use and to evaluate its psychometric properties.

## Methods

### Design

A methodological and developmental design was undertaken in two phases to construct and test this new instrument: (1) a panel of experts evaluated a pool of items using a content validation process, and (2) a cross-sectional survey was conducted to assess the validity and reliability of the developed instrument.

### Settings and Participants

Five outpatient diabetes clinics, two in northern Taiwan and three in southern Taiwan, were used as the settings for this study. Convenience sampling was used to recruit participants using the following inclusion criteria: (1) over 20 years old; (2) diagnosed with either type 1 or type 2 diabetes at least 12 months before the interview; (3) use of CTs for at least 3 months; and (4) sufficient cognitive and physical ability to understand and respond to the survey questions. Posters detailing the study were displayed in the clinics to inform potential participants. The research assistants approached patients during their clinic visits, explained the study purpose, and described the details involved in participation. The research assistants screened potential participants against the eligibility criteria and excluded those with severe physical or cognitive impairments that would prevent or hinder their ability to respond to the survey.

### Instrument Development

In developing the benefits-risks scale for CTs in diabetes care, the research team referenced H.-Y. Chang et al. (2021) and H.-Y. Chang, Yang, et al. (2023), which provide a decision algorithm for health care professionals to make optimal choices when engaging with patients with diabetes about the benefits and risks of using CTs. These studies informed the scale development process in this study, ensuring the tool is grounded in a consensus-driven understanding of key diabetes management considerations.

After considering the advantages and disadvantages of CT use from the perspective of health care professionals, the research team designed items to measure patient awareness and knowledge of CT use in diabetes, applying the applicable algorithm criteria for evaluating and managing CT use. A panel of 10 experts with expertise in diabetes, including academics (2), physicians (2), nurses (2), patients (2), a pharmacist (1), and a dietitian (1), used the content validity index to assess the quality and relevance of each item. Level of agreement with each item was assessed using a 4-point ordinal scale (1=*not relevan*t, 2=*somewhat relevant*, 3=*quite relevant*, and 4=*highly relevant*). The content validation process involved two evaluation rounds. In the first, most of the statements were identified as relevant and representative of the intended construct, achieving a content validity index of .88. However, two of the statements did not reach the threshold due to similarities between measuring, respectively, the concepts of “diabetes education” and “professional consultation” and the concepts of “support from health care professionals” and “support from institutions.”

The research team used this feedback to refine the items, resulting in a 16-item Understanding the Benefits-Risks of CT Use in Diabetes Scale before proceeding with further psychometric validation. The item stem in Chinese may be translated into English as: “How aware/knowledgeable are you? How well do you understand?” Respondents are asked to answer each item using a 4-point scale (1=*very unaware*, 2=*unaware*, 3=*aware*, 4=*very aware*). The total scale score ranges between 16 and 64, with higher scores indicating better understanding of the benefits and risks of using CTs in diabetes care.

### Data Collection

Survey data were collected between October 2022 and March 2023. In addition to the Understanding the Benefits-Risks of CT Use Scale, the following sociodemographic and clinical data were collected from the participants: age, gender, educational level, marital status, employment status, duration of diabetes, clinical visits, current diabetes treatment, and clinical laboratory results. Also, three single-item questions were used to measure (1) disclosure of CT use to health care professionals, (2) knowledge of herbal medicine ingredients, and (3) sources of information about CTs (H.-y. A. Chang et al., 2011) using the following respective questions: (1) “Have you told your doctor/nurse about your use of CTs?,” (2) “Did you know the ingredients of your herbal medicine at the time you were taking it?,” and (3) “Who is the primary decision-maker regarding which types of CT to use?”

To measure participant perceptions regarding the degree of control they have over their diabetes and their readiness for change, the 10-item Chinese Diabetes Empowerment Scale was also administered (Shiu et al., 2012). Patient empowerment refers to equipping patients with the knowledge, skills, and confidence necessary to gain more control over decisions and actions affecting their health and care (World Health Organization Regional Office for Europe, 2013). Therefore, this scale was chosen as the criterion-related validity measure as it aligns with the focus of the developed scale on understanding and managing the benefits and risks of using CTs. The participants responded to each item on the Chinese Diabetes Empowerment Scale using a 4-point Likert scale of agreement to facilitate direct comparability with the scale developed in this study. Higher scale scores indicate higher perceived control to self-direct and improve health. Shiu et al. reported the Cronbach’s α for internal consistency for the Chinese Diabetes Empowerment Scale as .77, and principal axis factor analyses supported a 5-factor structure (overcoming barriers, determining suitable methods, achieving goals, obtaining support, and coping).

### Data Analysis

All statistical analyses in this study were conducted on IBM SPSS Statistics 28.0 (IBM Corp., Armonk, NY, USA), and confirmatory analysis using structural equation modeling (SEM) was performed on Analysis of Moment Structures (AMOS), version 24.0. Descriptive statistics were used to describe participant sociodemographic and clinical characteristics. Other comparative analyses included *t* tests, one-way analysis of variance, and Pearson’s correlation for criterion-related validity.

Construct validity testing began with an exploratory factor analysis using principal axis factoring as the extraction method and varimax rotation (an orthogonal rotation technique). The Kaiser method, which considers eigenvalues >1, and the communalities of the varimax orthogonal rotation were then used to determine the number of factors or scale dimensions. Items with coefficients <0.4 were excluded to improve the interpretability of the results (Braeken & van Assen, 2017).

Confirmatory factor analysis was conducted using SEM to further assess the construct validity of the developed scale. The maximum likelihood estimation robust extraction method was employed to estimate parameter values. Convergent validity was displayed by composite reliability and average variance extracted (AVE), with recommended values of ≥0.7 and ≥0.5, respectively (Hair et al., 2022). Discriminant validity was used to assess whether the square root of the AVE for each construct was greater than the correlation coefficients between that construct and other constructs (Hair et al., 2022). To evaluate the overall fit of the model, the following six indices were assessed: the ratio of χ^2^ and degrees of freedom (<5), the goodness-of-fit index (GFI >.9), adjusted goodness-of-fit index (AGFI >.9), normed fit index (NFI >.9), comparative fit index (CFI >.9), and root mean square error of approximation (RMSEA < .08).

Cronbach’s alpha coefficient (α>.7) was used to estimate the within-scale consistency of the responses to scale items under the assumption of unidimensionality (LoBiondo-Wood & Haber, 2010), while composite reliability (>.8) was used to determine the internal consistency of indicator variable loadings on the latent variable without assuming unidimensionality and while considering the variance in factor loadings of the items (Hair et al., 2022).

### Ethical Considerations

Ethical approval for this study was obtained from the institutional review board of the Chang Gung Medical Foundation (No. 202102201B0) on January 10, 2022. An information sheet explaining the purpose of the research and research activities, describing the benefits of participation, assuring that data would be anonymized and confidentiality would be maintained, and stating that participation was wholly voluntary was given to potential participants. The participants were allowed to withdraw or terminate their participation at any point in the study without facing adverse consequences or jeopardizing their well-being. Data collection began after participants had provided written consent.

## Results

### Participants’ Characteristics

Ages of the 307 participants ranged from 25 to 94 years (Table 1); most (95.7%) had type 2 diabetes; most were female, married, had at least a high school education, were unemployed or retired, had been living with diabetes for over a decade, and visited diabetes specialist clinics at least once every 3 months. No statistical differences were found between scale scores and, respectively, gender, marital status, employment status, duration of diabetes, fasting plasma glucose, glycated hemoglobin, and frequency of clinic visits. However, scale scores were significantly higher among those participants with a high school or higher level of education.

**Table 1:** Associations Between Demographic and Clinical Characteristics and, Respectively, Scores on the Understanding the Benefits-Risks of Complementary Therapy Use Scale (*N*=307)

	Participants	Scale Score		
Characteristic	*M* (*SD*)	*M* (*SD*)	*r*	*p*
Age (year)	64.60 (10.99)	41.63 (7.10)	−.108	.059
Duration of diabetes (year)	11.21 (8.95)		.046	.419
Fasting plasma glucose (mg/dL)	141.82 (50.76)		−.034	.574
HbA1c	7.43 (1.61)		.013	.826
Empowerment scale	31.10 (5.05)		.426	<.001
	*n* (%)	*M* (*SD*)	*t*	*p*
Gender			−1.410	.160
Male	120 (39.1)	40.92 (7.47)		
Female	187 (60.9)	42.09 (6.83)		
Marital status			−1.142	.255
Single	30 (9.8)	41.48 (7.09)		
Married	277 (90.2)	43.03 (7.15)		
Highest level of education			−2.230	.026
<High school	115 (37.5)	40.57 (5.30)		
≥High school	192 (62.5)	42.26 (7.93)		
Employment status			1.415	.158
Employed	112 (36.5)	42.38 (7.49)		
Unemployed	195 (63.5)	41.19 (6.85)		
Frequency of clinical visits			0.241	.809
≤Monthly	41 (13.4)	41.88 (6.55)		
≥Every 3 months	266 (86.6)	41.59 (7.19)		

*Note.* HbA1c=glycated hemoglobin; Scale=The Understanding the Benefits-Risks of CT Use in Diabetes Scale.

### Validity Analysis

#### Construct validity analyses

The relatively high Kaiser–Meyer–Olkin (KMO) value of .886 and the significant result from the Bartlett test (*p* < .001) indicate the data were appropriate for conducting exploratory factor analysis. Principal component analysis structured the 16 items into four factors, each associated with an eigenvalue exceeding 1. The four factors were then labeled as the following dimensions: (1) risk assessment of CT use for patients’ medical condition (four items), (2) benefit-risk assessment of CTs (five items), (3) suitability of CT use (five items), and (4) support from health care professionals (two items).

**Figure 1:**
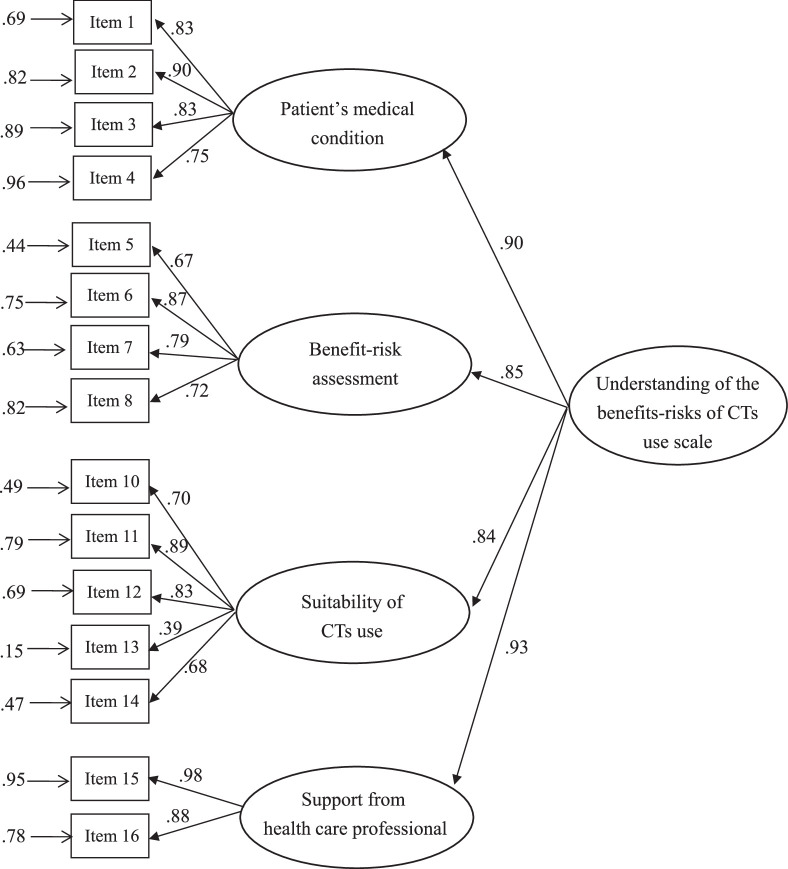
Multidimensional Model for the Understanding of the Benefits-Risks of Complementary Therapies (CTs) Use Scale.

The SEM showed all items had acceptable absolute skewness values. After adjusting the model to meet minimum threshold values for the goodness-of-fit indices and observing the covariance of modification indices, one item was found to have high multicollinearity with two other items, leading to its deletion from the model. The factor weights with corresponding latent variables in the final model were all >.60 (Figure 1). The factor loadings of all items exceeded the minimum threshold of .5, and the average variance extracted (AVE) indicators ranged from .52 to .93, demonstrating an acceptable level of convergent validity (Table 2). Each value was higher than the minimum threshold, indicating that all observed variables in the scale effectively aggregated on their respective factors. The model met all goodness-of-fit indices (GFI=.90, AGFI=.90, CFI=.94, NFI=.926, RMSEA=.08, and χ^2^ /*df*=3.14). The square root of the AVE for each construct was higher than the correlation coefficients between other constructs, supporting discriminant validity (Table 3).

**Table 2:** The Convergent Validity of the Benefits-Risks of Complementary Therapy (CT) Use Scale

	Parameter Estimation				Convergent Validity		
Dimension/Item	Estimate	*SE*	CR	*p*	Standardized Coefficient	CR	AVE
F1 Patient’s medical condition						.90	.69
Item 1 Overall health status	1.00				.83		
Item 2 Diabetes condition details	1.06	0.06	19.72	<.001	.91		
Item 3 Laboratory data	0.95	0.06	16.73	<.001	.83		
Item 4 Diabetes-related complications	0.83	0.06	14.55	<.001	.75		
F2 Benefit-risk assessment of CTs						.85	.59
Item 5 Efficacy of CTs	1.00						
Item 6 Quality standards of CTs	1.51	0.12	12.71	<.001			
Item 7 Constituents and ingredients of CTs	1.30	0.11	11.73	<.001			
Item 8 Potential side effects of CTs	1.13	0.11	10.77	<.001			
F3 Suitability of CTs use						.84	.52
Item 10 Purposes of CT use	1.00				.70		
Item 11 Recommended dosage of CT use	1.29	0.10	13.17	<.001	.89		
Item 12 Administration routes of CT use	1.12	0.09	12.53	<.001	.83		
Item 13 Cost and affordability of CTs	0.57	0.09	6.43	<.001	.39		
Item 14 Integration with conventional medicines	0.95	0.09	11.18	<.001	.69		
F4 Support from health care professionals						.93	.93
Item 15 Benefit-risk profile with HCP guidance	1.00				.98		
Item 16 Support and advice from HCP	0.93	0.06	16.47	<.001	.88		

*Note*. SE=standard error; CR=critical ratio; AVE=average variance extracted; HCP=health care professional.

**Table 3:** The Discriminant Validity of the Developed Scale

Construct	AVE	F1	F2	F3	F4
F1 Patient’s medical condition	.689	.830			
F2 Benefit-risk assessment of CTs	.586	.710	.766		
F3 Suitability of CT use	.519	.500	.543	.720	
F4 Support from health care professionals	.866	.433	.566	.433	.931

*Note.* AVE=The square root of average variance extracted.; CTs=complementary therapies.

#### Correlates with other measures

The scores of the Diabetes Empowerment Scale were found to relate significantly to the Understanding the Benefits-Risks of CT Use Scale (Table 1), with each of its four dimensions (*r*=.285–391, *p* < .001) also supporting the convergent validity of the construct. Furthermore, the Understanding the Benefits-Risks of CT Use Scale was identified as significantly related to the disclosure of CTs to health care professionals, knowledge of herbal medicine ingredients, and sources of information on CTs (Table 4).

**Table 4:** Comparison of Complementary Therapy (CT) Use Characteristics With Scores on the Understanding the Benefits-Risks of CT Use Scale (*N*=307)

	Participants	Scale Score		
Characteristic	*n* (%)	*M* (*SD*)	*t/F*	*p*
Reasons for usage			2.623	.074
➀ Not in favor of CTs	12 (3.9)	43.67 (5.85)		
➁ Belief in CTs	247 (80.5)	41.17 (7.04)	➂>➁	.041
➂ Recommended by HCP	48 (15.6)	43.46 (7.42)	−2.284	
Decision			1.511	.132
Individual	261 (85.0)	41.89 (6.89)		
Others	46 (15.0)	40.17 (8.11)		
Medicine compliance			0.320	.749
No change	303 (98.7)	41.64 (7.13)		
Reduce or stop	4 (1.3)	40.17 (8.11)		
Knowing the ingredients of CTs			7.998	<.001
No	122 (39.7)	39.23 (5.91)		
Yes	185 (60.3)	45.26 (7.23)		
Sources of information on CTs			2.493	.013
Professionals	54 (17.6)	43.80 (6.61)		
Nonprofessionals	253 (82.4)	41.17 (7.13)		
Disclosure of CAM			−5.444	<.001
No	214 (69.7)	40.24 (6.60)		
Yes	93 (30.3)	44.83 (7.20)		

*Note*. HCP=health care professionals; CAM=complementary and alternative medicine; Scale=The Understanding the Benefits-Risks of CT Use in Diabetes Scale.

### Reliability Analysis

The Cronbach’s α coefficients of the four dimensions ranged from .821 to .926, with a total scale internal consistency of .912. The SEM-based composite reliability of the scale’s four dimensions ranged from .836 to .928 (Table 2).

## Discussion

The results of this study demonstrate the adequate reliability and validity of the newly developed Understanding the Benefits-Risks of CT Use Scale and the dependability of this scale in capturing patient understanding of the risks and benefits of using CTs in diabetes management. Moreover, none of the four dimensions of this scale was found to exhibit bias in terms of gender, marital status, or health characteristics. Furthermore, existing instruments such as the International Complementary and Alternative Questionnaire, the Holistic CAM Questionnaire, and the CAM Health Belief Inventory, which focus primarily on general usage patterns, overall beliefs, and holistic health perspectives, are unable to assess respondents’ nuanced understandings of the benefits and risks of CTs in the context of diabetes management. The scale developed in this paper was designed specifically to evaluate the depth of patient knowledge regarding the benefits and risks of CTs, including knowledge of potential interactions with conventional diabetes treatments, possible side effects, and the scientific evidence supporting various CTs. In providing a detailed knowledge-based assessment, this scale may be used to identify gaps in understanding that may influence CT safety and efficacy.

The four dimensions of the Understanding the Benefits-Risks of CT Use Scale include (1) risk assessment of CT use for patients’ medical condition, (2) benefit-risk assessment of CTs, (3) suitability of CT use, and (4) support from health care professionals. The four items of the first dimension focus on patient understanding of their health status while using CTs, their understanding of potential risks in managing and controlling diabetes, risks of altering medical laboratory data, and the potential development of disease-related complications. This assessment provides valuable information about individual health profiles and helps health care professionals recognize their patients’ understanding and knowledge of potential risks and contraindications associated with CT use (H.-Y. Chang et al., 2021). Notably, demographic and clinical factors are essential considerations in determining the suitability and safety of CT use.

The four items in the second dimension are designed to assess patient understanding and knowledge of whether their CTs are of adequate quality and effectiveness, as well as patient awareness of the contents and potential side effects of these items. This information assists health care professionals in assuring patients sufficiently understand the safety, reliability, effectiveness, and side effects/interactions with conventional medicine of CT medicaments taken (H.-Y. Chang, Mao, et al., 2023; Thikekar et al., 2021). Items associated with significant clinical effectiveness, quality-regulated manufacturing, safe and disclosed ingredients, and a favorable safety profile with minimal potential risks and no significant interactions with conventional medicine may be considered suitable as a safe, reliable, and effective CT for diabetes management.

The five items in the third dimension address patient awareness of the suitability of CT use by focusing on the route of administration, purpose of usage, dosage, costs and financial burden, and treatment compliance. For health professionals, this information improves the overall benefit-risk profile of CTs by providing insights into patient understanding of CT safety, alignment with treatment goals, dosage manageability, financial feasibility, and patient compliance (H.-Y. Chang et al., 2021; Ng et al., 2021). Based on the scores in this dimension, health care professionals can conduct targeted discussions with patients regarding the safety of CTs (noninvasive vs. invasive) and their compatibility with conventional medicine in the context of diabetes management. Also, these scores help health care providers evaluate and recommend appropriate CT medicament dosages. Finally, health care professionals will learn whether their patients understand the affordability of applying CTs in their diabetes management strategy.

Finally, the two items in the fourth dimension assess whether patients recognize or are aware of the level of support available from health care professionals regarding CTs. Health care professionals play a vital role in fostering open dialogue with patients with diabetes about their CT use. Mutual communication between patients and health care professionals ultimately helps patients make better-informed decisions when integrating CTs into their diabetes care (Stie et al., 2020).

The new scale developed in this paper facilitates the granular analysis of patient understanding of the risks and benefits associated with the CTs they are currently using in their diabetes management program. Scale scores were found to relate significantly with variables that collectively pertain to the concept of health literacy, such as obtaining information about CTs from health care professionals, feeling empowered, and disclosing CT use to health care professionals. Higher health literacy is associated with a better ability to access, comprehend, and apply information and services, critical to making better-informed decisions and initiating appropriate health-related actions (Lee & Son, 2022; Pourhabibi et al., 2022). In individuals who want to use CTs, health literacy helps empower them to seek reliable sources, critically evaluate information, and engage in effective communication with health care professionals. This specificity is crucial for health care professionals to offer precise guidance and ensure patient safety.

### Limitations

The convenience sample used in this study introduced the potential for sampling bias. However, a comparison of the sample in this study with samples covered in a systematic review by Alzahrani et al. (2021), which examined the global prevalence of CT use in patients with diabetes, showed similar gender ratios and lengths of time since diabetes diagnosis. This comparative analysis strengthens the generalizability and external validity of the findings of this study to larger populations of patients with diabetes using CTs. To address the potential influence of social desirability bias on this study, during the construct validation process, confirmatory factor analysis was used to assess convergent and discriminant validity by examining the relationships between scale scores and multiple variables, thereby ensuring the robustness of the instrument and minimizing the potential impact of response bias.

### Implications

A scale that can accurately assess patient understanding of the risks and benefits of the use of CTs in diabetes management programs helps identify areas of insufficient understanding/knowledge. The results are hoped to guide future researchers in targeting knowledge gaps and addressing concerns about CTs in education and communication. In addition, using this scale in clinical settings is expected to facilitate improved communication between health care professionals and patients about CT-related regulations and safety guidelines. Furthermore, by identifying specific gaps in patient understanding and knowledge, health care professionals can deliver more individualized and targeted educational interventions. Communicating the potential benefits and risks associated with CTs may be expected to promote informed decision-making and patient empowerment. Overall, the scale developed and validated in this study is expected to promote the safer and more effective application of CTs in diabetes management.

### Conclusions

The Understanding the Benefits-Risks of CT Use Scale is a specialized assessment tool that may be used by health care professionals to evaluate the understanding and knowledge of their patients regarding the benefits and risks associated with using CTs in their diabetes management program. The results foster better-informed decision-making and empowerment with regard to using CTs in diabetes management. The new instrument provides a valuable and reliable tool that health care professionals and researchers may use to address patient knowledge gaps and enhance patient education, ultimately improving patient-centered health care strategies and ensuring patient safety and well-being.
